# A MEK inhibitor arrests the cell cycle of human conjunctival fibroblasts and improves the outcome of glaucoma filtration surgery

**DOI:** 10.1038/s41598-024-52359-y

**Published:** 2024-01-22

**Authors:** Jinhee Lee, Megumi Honjo, Makoto Aihara

**Affiliations:** Department of Ophthalmology, Graduate School of Medicine, The University of Tokyo, 7-3-1 Hongo Bunkyo-ku, Tokyo, 113-8655 Japan

**Keywords:** Eye diseases, Molecular medicine

## Abstract

Better agents are needed to improve glaucoma filtration surgery outcomes compared to current ones. The purpose of this study is to determine whether mitogen-activated protein kinase kinase (MEK) inhibitors can effectively arrest the cell cycle of human conjunctival fibroblasts (HCFs) and inhibit the formation of fibrosis and scarring following glaucoma filtration surgery. A cell counting kit‑8 assay revealed that the MEK inhibitor PD0325901 exhibited concentration-dependent growth inhibition of HCFs. Quantitative PCR, immunocytochemistry, and western blotting demonstrated decreased expression of proliferating cell nuclear antigen (PCNA) and cyclin D1 and increased expression of p27 in HCFs treated with PD0325901. Flow cytometry indicated that PD0325901 arrested the cell cycle of HCFs in the G0/1 phase. The cell-migration assay showed that HCF migration rate was significantly suppressed by PD0325901 exposure. Rabbits were divided into PD0325901-treatment and control groups, and glaucoma filtration surgery was performed. Although intraocular pressure did not differ between PD0325901-treatment and control groups, bleb height was greater in the treatment group. Histopathological evaluation revealed that fibrotic changes were significantly attenuated in the PD0325901-treatment group compared to the control group. In conclusion, the MEK inhibitor impedes HCF proliferation via cell-cycle arrest and may be beneficial for glaucoma filtration surgery by reducing bleb scarring.

## Introduction

Glaucoma filtration surgery is the standard procedure for glaucoma patients whose intraocular pressure (IOP) cannot be adequately controlled with medication^1^. Glaucoma filtration surgery facilitates the drainage of aqueous humor under the conjunctiva. However, excessive postoperative scar formation can lead to the loss of subconjunctival space, resulting in surgical failure^1^. Currently, 5-fluorouracil (5-FU) and mitomycin-C (MMC) are applied during the perioperative period to inhibit scar formation^2^. These agents are commonly used for controlling postoperative IOP, however, they can induce complications such as bleb leakage, hypotony, avascularized bleb, and endophthalmitis^3^. Consequently, there is a need for drugs that can safely lower IOP with fewer side effects than 5-FU and MMC, or can improve the benefits of existing anti-scarring drugs.

Existing evidence indicates that several factors in the aqueous humor contribute to postoperative subconjunctival fibrosis^4^. Tumor growth factor (TGF)-β is a major aqueous mediator that induces the contraction, proliferation, and migration of human conjunctival fibroblasts (HCFs), and has been used in in vitro glaucoma filtration surgery experiments^5^. However, in a previous study, administration of anti-TGF-β2 antibody during trabeculectomy did not improve surgical outcomes over a placebo^6^, implying that suppression of TGF-β alone is inadequate.

Fibroblasts in most tissues exist in a quiescent phase; however, surgical intervention activates tissue-repair mechanisms, leading to cell-cycle progression^7^. Excessive wound healing responses result in fibrotic scar formation^8^; therefore, strategies to regulate the cell cycle may be beneficial for successful glaucoma filtration surgery. For instance, fetal bovine serum (FBS) induces HCF proliferation and has been used in several studies of HCF cell-cycle regulation^9,10^.

Mitogen-activated protein kinase kinase (MEK) inhibitors have demonstrated efficacy in both arresting the cell cycle^11^ and preventing fibrosis through a rapidly accelerated fibrosarcoma (Raf)/MEK/extracellular signal-regulated kinase (ERK) pathway^12,13^. Recently, MEK inhibitors have been applied in the treatment of melanoma^14^, and clinical trials using MEK inhibitors have been conducted for lung cancer^15^ and thyroid carcinoma^16^. The MEK inhibitor U0126 suppresses TGF-β1-induced myofibroblast transdifferentiation in HCFs^17^. However, the effects of MEK inhibitors on HCF cell-cycle arrest and their application in glaucoma filtration surgery in vivo have not been investigated.

In this study, we investigated the effect of a MEK inhibitor on cell-cycle arrest in serum-stimulated HCFs, and evaluated its treatment efficacy in a rabbit model of glaucoma filtration surgery.

## Results

### Effects of PD0325901 on toxicity and proliferation

**Figure 1:**
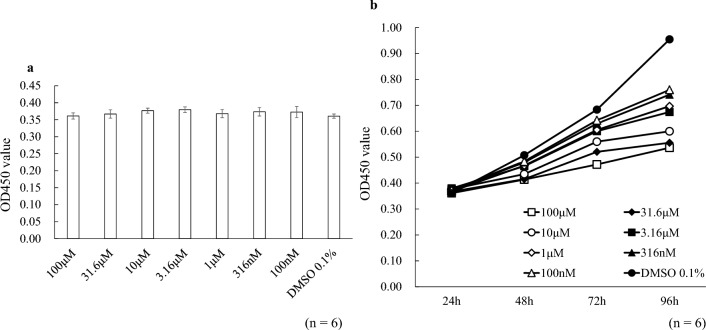
Effects of PD0325901 on toxicity and proliferation. (**a**) To assess toxicity, HCFs were incubated for 24 h with PD0325901 (100 nM–100 µM or 0.1% DMSO, after which the OD450 was measured using the CCK-8 assay. No significant differences were observed among the groups. (**b**) To evaluate proliferation, HCFs were incubated for up to 96 h with PD0325901 or 0.1% DMSO, after which the OD450 was measured using the CCK-8 assay. The points in this figure show the average OD450 at each PD0325901 concentration or 0.1% DMSO. PD0325901 inhibited HCF proliferation in a concentration-dependent manner.

PD0325901 (Mirdametinib; FUJIFILM Wako Pure Chemical, Osaka, Japan), a selective MEK inhibitor, was used. First, the 24-h toxicity of PD0325901 in HCFs was confirmed using the cell counting kit‑8 (CCK‑8; Dojindo Molecular Technologies, Inc., Kumamoto, Japan) assay (Fig. 1). The OD450 of cells exposed to PD0325901 did not differ from that of the 0.1% dimethyl sulfoxide (DMSO) control. However, 96 h of PD0325901 exposure showed a concentration-dependent HCF growth-inhibition effect. As concentrations of 1 µM or higher showed significant growth inhibition at all time points after 48 h (P < 0.041), and there was no difference in OD450 up to 72 h at concentrations of 100 nM and 316 nM compared to DMSO (P > 0.097), we used 1 and 10 µM PD0325901 in subsequent experiments.

### Effects of PD0325901 on mRNA expression

Proliferating cell nuclear antigen (PCNA) plays a crucial role in the cell cycle, particularly in DNA replication and repair^18^. PCNA is an auxiliary protein for DNA polymerase that reaches maximal expression during the S phase of the cell cycle. Cyclin D1 plays a central role in the regulation of proliferation, linking the extracellular signaling environment to cell cycle progression^19^. It is involved in the transition from the G1 phase to the S phase of the cell cycle, promoting cell cycle progression by binding to and activating cyclin-dependent kinase (CDK) 4/6, which in turn phosphorylates and inactivates the retinoblastoma protein, allowing the cell to enter the S phase. p27 is a key regulator of the cell cycle, exerting multiple functions in cell cycle control, apoptosis, epigenetic modification, and transcriptional regulation^20^. Its inhibitory effect on the cell cycle is mediated by the inhibition of CDKs, thereby preventing the progression of the cell cycle from the G1 phase. Since the decrease in cyclin D1 and PCNA, and the increase in p27 could indicate an effect of PD0325901 on cell cycle arrest in the G1 phase, these markers were investigated in this study.

**Figure 2:**
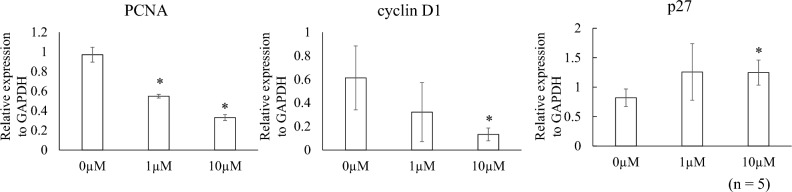
Effects of PD0325901 on mRNA expression. PCNA expression decreased after exposure to 1 and 10 μM PD0325901 compared to 0.1% DMSO (P < 0.001). Compared to controls, cyclin D1 expression decreased and p27 expression increased after exposure to 10 μM PD0325901. *P < 0.05 vs. 0.1% DMSO control.

Figure 2 shows the Quantitative real-time PCR (qPCR) results. PCNA expression was significantly lower in the 1- and 10-µM PD0325901 treatment groups than in the 0.1% DMSO control (P < 0.001). Compared to controls, cyclin D1 expression was significantly lower and p27 expression was significantly higher after treatment with 10 µM PD0325901 (P = 0.026, 0.013, respectively).

### Effects of PD0325901 on the expression of protein level

**Figure 3:**
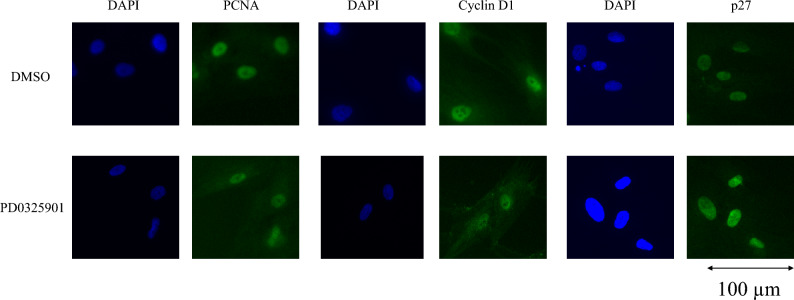
Effects of PD0325901 on immunocytochemistry. Expression of PCNA and cyclin D1 was lower, and p27 expression was higher, in PD0325901-treated HCFs compared to DMSO-treated HCFs.

**Figure 4:**
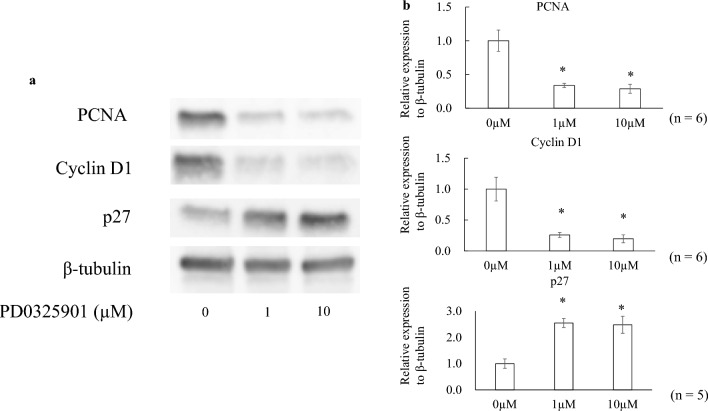
Effects of PD0325901 on Western blotting. (**a**) Representative bands from the western blotting assay. Results are expressed relative to the loading control (β-tubulin). (**b**) Western blotting revealed decreased PCNA and cyclin D1 expression, and increased p27 expression, after exposure to 1 and 10 µM PD0325901 exposure compared to controls (P < 0.001). *P < 0.05 vs. 0.1% DMSO control. The original blots are presented in Supplementary Figs. 1 and 2. Samples from the same experiment were used, and gels and blots were processed simultaneously.

The immunocytochemistry results are shown in Fig. 3. Treatment with PD0325901 downregulated PCNA and cyclin D1 expression but upregulated p27 expression. Figure 4 shows the western blotting results. PCNA and cyclin D1 expression were significantly lower, and p27 expression significantly higher, in both the 1 and 10 µM PD0325901 treatment groups than in the 0.1% DMSO control (all P < 0.001).

### Effects of PD0325901 on cell cycle

**Figure 5:**
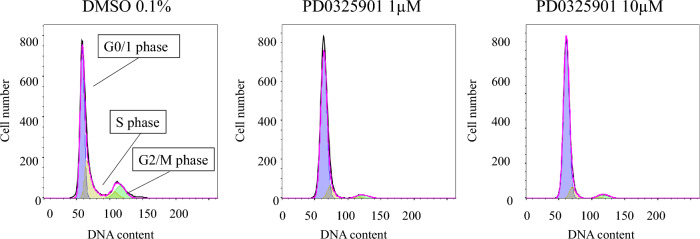
Effects of PD0325901 on cell cycle. Treatment with both 1 and 10 μM PD035901 increased the percentage of HCFs in the G0/1 phase and decreased the percentages of HCFs in the S and G2/M phases compared to the 0.1% DMSO control (all P < 0.008).

The percentage of HCFs in the G0/1 phase was higher, and those of the S and G2/M phases were lower, in both treatment groups versus controls (Table 1, Fig. 5, P < 0.008).

**Table 1:** Effect of PD0325901 on cell cycle of HCFs.

	DMSO	1 µM of PD0325901	10 µM of PD0325901
G0/G1 phase (%)	56.3 ± 1.7	84.9 ± 2.0	85.9 ± 2.1
S phase (%)	28.9 ± 1.3	9.0 ± 0.9	8.4 ± 0.7
G2/M phase (%)	10.2 ± 1.5	3.3 ± 0.8	4.9 ± 1.3

n = 4, mean ± SD.

### Effects of PD0325901 on migration assay

**Figure 6:**
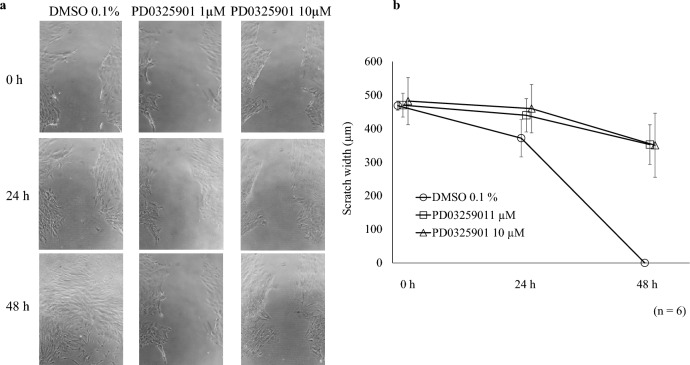
Effects of PD0325901 on migration assay. The cell-migration assay was performed on HCFs for 0, 24, and 48 h (n = 6). The baseline (0 h) represented the migration distance of cells with no stimulation. (**a**) The scratch width did not differ among the groups at 24 h. (**b**) At 48 h, all DMSO-group replicates showed reduced widths whereas all treatment replicates retained their widths; migration was significantly suppressed by 1 and 10 μM PD0325901 exposure at 48 h post-incubation compared to the DMSO control (P < 0.001).

Figure 6a shows typical images of the cell-migration assay. There were no differences in scratch width between the DMSO and PD0325901 groups at up to 24 h. However, at 48 h, the scratch disappeared in all controls but remained in all PD0325901 treatment samples; the scratch widths significantly differed between the treatment groups and controls (Fig. 6b, P < 0.001).

### Effects of PD0325901 on a glaucoma filtration model in rabbits

**Figure 7:**
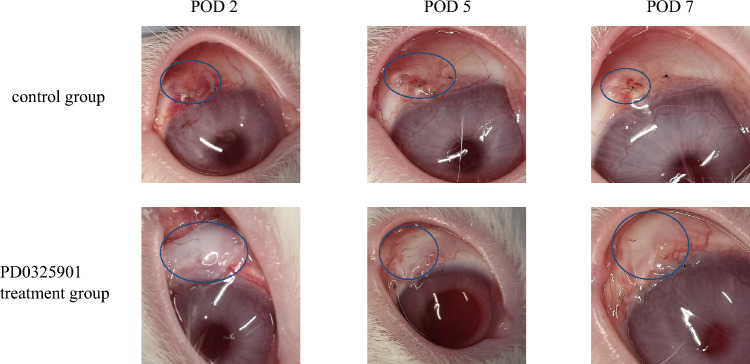
Representative photographs after glaucoma filtration surgery. Representative photographs of the postoperative conjunctival bleb at postoperative days 2, 5, and 7. Less hyperemia and diffuse blebs were observed in the PD0325901 group compared to the control group. The circle lines indicate the extent of the bleb.

**Figure 8:**
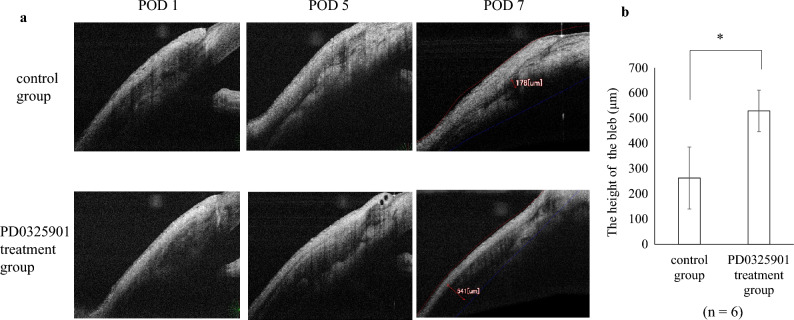
Anterior segment optical coherence tomography (AS-OCT) after glaucoma filtration surgery. (**a**) The space in the bleb in the PD0325901 treatment group was better maintained compared to that in the DMSO control group. (**b**) Bleb heights on postoperative day 7 were significantly greater in the treatment group than in controls (P = 0.003).

**Figure 9:**
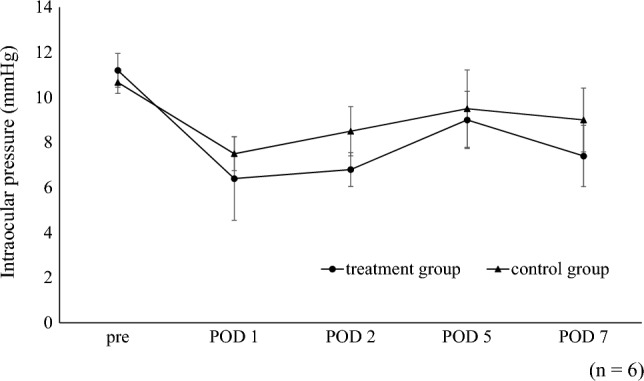
Intraocular pressure after glaucoma filtration surgery. No significant differences in IOP were observed between the treatment and control groups at any time point.

Representative photographs after glaucoma filtration surgery are shown in Fig. 7. The PD0325901 treatment group showed less hyperemia and more diffuse blebs than the control group. It also had a greater average bleb height on postoperative day 7 (Fig. 8). Although IOP tended to be lower in that group, it did not significantly differ from the control on any of the measurement dates (Fig. 9). No differences were observed between the treatment and control groups in slit-lamp and fundus examinations.

**Figure 10:**
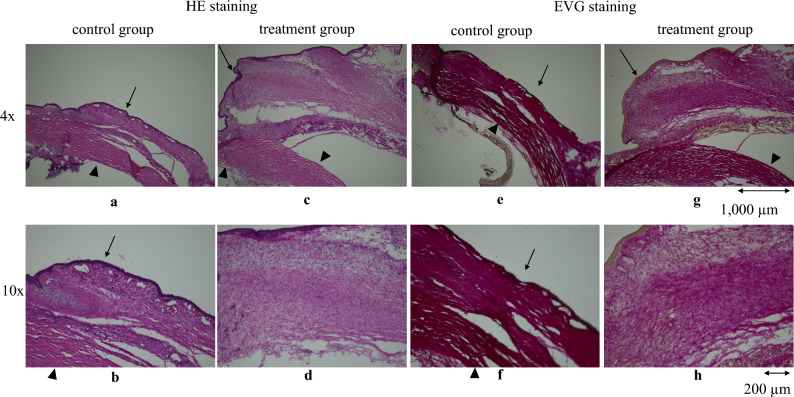
Histopathologic evaluation of blebs. HE staining of eye tissue revealed dense cells and fibrotic scarring in the control group (**a,b**) and relatively sparse cells and milder scar formation in the treatment group (**c,d**). EVG staining revealed dense collagen deposition and scar formation in the conjunctiva and sclera of the control group (**e,f**), whereas collagen deposition was faintly stained, conjunctival tissue was loose, and the subconjunctival space was maintained in the treatment group (**g,h**). Magnification: ×4 for (**a,c,e**,**g**); ×10 for (**b,d,f**,**h**). Arrows: the conjunctiva, arrow heads: the sclera.

Hematoxylin and eosin (HE) staining revealed densely packed cells in the bleb area of controls, suggestive of fibrosis (Fig. 10a, b). Conversely, in the treatment group, the cells were relatively sparse and scar formation was suppressed (Fig. 10c, d). Elastica van Gieson (EVG) staining revealed densely stained collagen deposits and scar formation in both the conjunctiva and sclera of controls (Fig. 10e, f). By contrast, in the PD0325901 treatment group, collagen deposits were faintly stained, conjunctival tissue had a loose appearance, and the subconjunctival space was preserved (Fig. 10g, h).

## Discussion

We investigated whether PD0325901, a MEK inhibitor, could arrest the HCF cell cycle and prevent scar formation after glaucoma filtration surgery. The CCK-8 assay revealed concentration-dependent growth inhibition of HCFs by PD0325901. Immunocytochemistry, qPCR, and western blotting showed that PD0325901 downregulated cyclin D1 and PCNA expression, and increased p27 expression, in HCFs. Flow cytometry indicated that it arrested the HCF cell cycle in the G0/1 phase, while the migration assay revealed that it inhibited migration of HCFs in vitro, implying an inhibitory effect on wound healing. In a rabbit model of glaucoma filtration, PD0325901 effectively maintained bleb volume and prevented fibrotic scarring.

ERK1/2 is ubiquitously expressed and is part of the Raf/MEK/ERK signal transduction cascade^21^. This cascade plays a crucial role in cell-cycle progression, cell migration, proliferation, and transcription^21^. The in vitro experimental results of our study reveal the effects of a MEK inhibitor in terms of arresting the HCF cell cycle and inhibiting cell proliferation.

MEK and p38 are involved in the mitogen-activated protein kinase (MAPK) cascade^22^. p38 is activated by oxidative stress and inflammatory cytokines, and is involved in the regulation of cellular responses such as inflammation, apoptosis, and differentiation^22^. It has been shown that p38 inhibitors suppress myofibroblast transdifferentiation of HCFs and are effective for treating a rabbit model of glaucoma filtration surgery^23,24^. The MEK cascade differs from p38 in that it is activated by growth factors and hormones and is involved in regulating cell proliferation^22^. Wen et al*.* demonstrated that another MEK inhibitor, U0126, suppressed myofibroblast transdifferentiation of HCFs, as well as TGF-β1-induced phosphorylation of Smad2/3, p38/MAPK, and ERK1/2^17^. Thus, MEK inhibitors, as with p38 inhibitors, may effectively regulate postoperative fibrosis after glaucoma filtration surgery through the modulation of scar formation via the MAPK cascade.

In addition to the above-described mechanisms via signaling-pathway suppression, we found that PD0325901 attenuated fibrosis by arresting the HCF cell cycle. PD0325901 significantly blocked the cell cycle and prevented cell proliferation but did not cause HCF cell death. It did not show high cytotoxicity compared to other antifibrotic drugs, such as MMC, implying that it can be used safely in combination with other antifibrotic drugs in glaucoma surgery. We also showed the effective suppression of fibrosis in a rabbit model of glaucoma filtration surgery via subconjunctival injection of PD0325901. Regarding the relationship between cell-cycle regulation and the suppression of fibrosis after filtration surgery, a recent study also showed that subconjunctival injection of adrenaline during trabeculectomy or other filtration surgeries effectively suppressed the contractile properties of HCFs and inhibited fibroblast proliferation by blocking key cell-cycle genes^25^. MEK inhibitors have been widely used for the treatment of neoplastic disorders, with the expectation that they suppress tumor cell growth^14–16^, particularly in combination with other therapeutic agents. As currently used drug therapies, 5-FU and MMC effectively suppress postoperative fibrosis in most, but not all, cases. During the time course of postoperative fibrosis formation, the inhibition of multiple complex cellular interactions is essential and should be regulated. The administration of multiple inhibitors has the potential for synergic effects^26^. For example, we previously reported the effects of an mTOR inhibitor on HCF proliferation and suppression of fibrosis after filtration surgery in rabbits^27^. Meanwhile, a synergistic antiproliferative effect has been reported for the combined use of an mTOR inhibitor and MEK inhibitor in tumor cells^26^. Thus, the combination of several inhibitors may be useful for the safe and effective suppression of HCF proliferation.

5-FU inhibits the production of deoxythymidine monophosphate, which is essential for DNA synthesis, contributing to its cytotoxic effects^28^. MMC is an alkylating agent that crosslinks DNA, thereby directly causing DNA damage and interfering with DNA synthesis and cell division^1^. In contrast, MEK inhibitors target the MEK/ERK pathway, which is a key signaling pathway involved in cell proliferation and survival^11^. The use of DNA-damaging reagents such as MMC induces activation of ERK^29^, which has also been suggested to contribute to cell cycle re-entry after DNA damage-induced cell cycle arrest^30,31^. Hence, MEK inhibitors are expected to be useful as adjuncts to MMC. Further research is needed to explore this topic.

Although in our study IOP tended to be lower in the PD0325901 group, there were no significant differences between the treatment and control groups at any time points. In the rabbit strain used in this study, the baseline IOP was so low that any IOP-lowering effect via sustainable bleb formation with PD0325901 could not be verified. In a future study, we plan to use rabbit or other animal models of ocular hypertension to assess this effect.

This study had several limitations. First, we did not compare PD0325901 with currently used perioperative agents such as MMC or 5-FU. Second, the postoperative observation period was short. Because bleb failure in rabbits has been reported to occur within 10–14 days postoperatively without the use of antimetabolites, the observation period was set at 7 days^32^. Long-term observation is necessary for comparing the effect of PD0325901 and antimetabolites on scar formation. In addition, a sustained drug-delivery system may be useful and should be explored in the future. Third, the side effects of PD0325901 have not been adequately observed. MEK inhibitors can cause retinopathy and uveitis^33^. Vitreous injection of PD0325901 at concentrations much higher than those tested in this study causes retinopathy in rabbits^34^. Since there is a report of MEK inhibitor hindering wound healing of corneal epithelial cells^35^, corneal damage may need to be carefully monitored when using MEK inhibitor intraoperatively. Although we observed no adverse effects, additional studies are needed to determine the safe dose of PD0325901. Forth, two bands of different molecular weights are observed in the p27 membrane in the supplement figure. p27 isoform has been reported^36^, and bands at similar molecular weight positions was found in a previous report^37^. The results of this study may be due to the p27 isoform.

In summary, PD0325901 arrests the cell cycle of HCFs and may be beneficial in suppressing scar formation after glaucoma filtration surgery.

## Methods

### Cell culture and passage

Primary HCFs were obtained from human donor eyes and characterized as described previously^38^. The cells were cultured in Dulbecco’s modified Eagle’s medium (DMEM)/F-12 containing 10% FBS and antibiotic–antimycotic solution (100×) (Sigma-Aldrich, St. Louis, MO, USA) in a CO_2_ incubator at 37 °C. In all experiments, only cells from passages 3–6 were used. To arrest the cell cycle, HCFs were grown to confluence and starved for 48 h in DMEM/F-12 without FBS.

### Cell proliferation assay

The cytotoxicity and growth-inhibition effects of PD0325901 were assessed using a CCK-8 assay. After cell-cycle arrest, 1 × 10^4^ cells were replated in a 96-well plate in DMEM/F-12 medium with 10% FBS with or without PD0325901 (100 nM–100 µM dissolved in 0.1% DMSO or 0.1% DMSO in phosphate-buffered saline (PBS) in a CO_2_ incubator at 37 °C. After incubation for 24, 48, 72, or 96 h, 10 µL CCK‑8 solution was added to each well and incubated at 37 °C for 2 h. The absorbance at 450 nm (OD450) was measured using a multimode plate reader (Multi Microplate Reader, ARVOX3; Parkin Elmer, MA, USA).

### Quantitative PCR

Following serum starvation, 5 × 10^4^ cells were replated in a 24-well plate in DMEM/F-12 medium with 10% FBS with or without PD0325901 (1 or 10 µM). After incubation for 48 h, the cells were lysed using ISOGEN (NIPPON GENE Ltd., Tokyo, Japan), and isopropyl alcohol and chloroform were used to extract mRNA. qPCR was performed as described previously^27^. Primers were purchased from Hokkaido System Science (Hokkaido, Japan). The primer sequences in this study were referred to those used in previous reports^39–42^. The sequences of the PCR primers were: GAPDH: forward, 5′-GTCTCCTCTGACTTCAACAGCG-3′ and reverse, 5′-ACCACCCTGTTGCTGTAGCCA-3′; PCNA: forward, 5′-CCTGCTGGGATATTAGCTCCA-3′ and reverse, 5′-CAGCGGTAGGTGTCGAAGC-3′; p27: forward, 5′-TAATTGGGGCTCCGGCTAACT-3′ and reverse, 5′-TTGCAGGTCGCTTCCTTATTC-3′; and cyclin D1: forward, 5′-AGCTGTGCATCTACACCGA-3′ and reverse, 5′-GAAATCGTGCGGGGTCATTG-3′. The expression levels of GAPDH were used to normalize those of PCNA, p27, and cyclin D1.

### Immunocytochemistry

After serum starvation, 5 × 10^4^ cells were replated in 24-well plates with glass coverslips in DMEM/F-12 medium containing 10% FBS with 1 μM PD0325901 or 0.1% DMSO. Following 48 h incubation, the cells were fixed in ice-cold 4% paraformaldehyde for 15 min. Permeabilization was performed with 0.3% Triton X-100 for 5 min. For blocking, Blocking One Histo (Nacalai Tesque, Kyoto, Japan) was used for 30 min at room temperature. Immunocytochemistry was performed as described previously^27^. The cells were incubated at 4 °C overnight with primary antibodies. The primary antibodies were anti-PCNA (1:200; Sigma-Aldrich), anti-p27 (1:1000; Cell Signaling Technology, Danvers, MA, USA), and anti-cyclin D1 (1:1000; Cell Signaling Technology). The cells were washed with PBS and incubated with the secondary antibody (Alexa Fluor 488; 1:1000; Thermo Fisher Scientific, Waltham, MA, USA) for 60 min at room temperature. Nucleus staining was performed using 4 μg/mL of 4′,6-diamidine-2-phenylindole dihydrochloride solution (DAPI; FUJIFILM Wako Pure Chemical).

### Western blotting

After cell-cycle arrest, 1 × 10^6^ cells were replated in DMEM with 10% FBS with or without PD0325901 (1 or 10 µM) or 0.1% DMSO in a 60-mm dish, and incubated for 48 h. The cells were obtained using radioimmunoprecipitation assay buffer (RIPA buffer; Thermo Fisher Scientific K.K., Kanagawa, Japan) and protease inhibitors (Roche Diagnostics, Basel, Switzerland). A BCA Protein Assay Kit (Thermo Fisher Scientific K.K.) was used to ascertain the protein quantities in the supernatant. Western blotting was performed as described previously^27^. The following primary antibodies were incubated at 4 °C overnight: anti-PCNA (1:1000; Sigma-Aldrich), anti-p27 (1:1000; Cell Signaling Technology), anti-cyclin D1 (1:1000; Cell Signaling Technology), and anti-β-tubulin (1:1000; FUJIFILM Wako Pure Chemical). Horseradish peroxidase-conjugated secondary antibody (H goat anti-rabbit or anti-mouse IgG; 1:5000; Thermo Fisher Scientific) was incubated for 1 h at room temperature. An ImageQuant LAS 4000 mini-instrument (GE Healthcare, Chicago, IL, USA) was used to detect protein bands. WB Stripping Solution (Nacalai Tesque) was used to strip membranes of antibodies. The density of protein bands was measured using ImageJ ver. 1.53 (National Institutes of Health, Bethesda, MD, USA). Protein expression was quantified against that of β-tubulin.

### Cell cycle analysis

After serum starvation, 3 × 10^6^ cells were replated in DMEM with 10% FBS with or without PD0325901 (1 or 10 µM) or 0.1% DMSO in a 100-mm dish, and then incubated for 48 h. The cells were trypsinized, washed in PBS, and fixed with 70% ethanol at –20 ℃. Fixed cells were stained with 50 mg/mL propidium iodide. Flow cytometric analysis was performed using a BD FACSAria™ III cell sorter (BD Biosciences, San Jose, CA, USA). The percentage of HCF in each cell cycle was obtained by the Watson Pragmatic algorithm in FlowJo v10.

### Migration assay

Confluent HCFs in a 24-well plate were serum starved for 48 h. Each well was scratched crosswise with a pipette tip. After washing with PBS, the medium was changed to DMEM/F-12 with PD0325901 (1 or 10 µM) or 0.1% DMSO. Scratches were photographed at 0, 24, and 48 h after stimulation using a microscope (BZ-9000; Keyence Corporation, Osaka, Japan). The shortest distance between the edges of cells on either side of the scratch was measured using ImageJ ver. 1.53.

### Animals

This study was carried out in accordance with the Association for Research in Vision and Ophthalmology Statement for the Use of Animals in Ophthalmic and Vision Research, and was approved by the Institutional Animal Research Committee of the University of Tokyo. The research has been reported in accordance with ARRIVE guidelines. The right eyes of 12 male Japanese white rabbits (14–19 weeks old, 1.50–1.99 kg; Kitayama Labes Co., Ltd., Nagano, Japan) were used in the current study. Before the experiments started, every rabbit was allowed 6 days to acclimate. The rabbits were housed in a conventional animal room with a 12-h light/dark cycle, and temperature and humidity controls of 22 °C ± 3 °C and 55% ± 10%, respectively. The rabbits were given tap water and food ad libitum.

### Glaucoma filtration surgery in rabbits

All surgical procedures were conducted by JL. Rabbits were anesthetized with an intramuscular injection of ketamine hydrochloride (5 mg/kg body weight) and xylazine hydrochloride (5 mg/kg body weight). A fornix-based flap of the conjunctiva was made in the superior region. After sclerostomy, a fistula leading to the anterior chamber was made. The conjunctival flap was sutured using 10-0 nylon.

### Evaluation of the effect of PD0325901 in rabbits

The rabbits were randomly divided into a treatment group (n = 6) and a control group (n = 6). Photographs of the filtering bleb were captured using a digital camera. Slit-lamp and fundus examination were conducted. The height of the filtering bleb was measured using anterior segment optical coherence tomography (AS-OCT; RS-3000; NIDEK, Inc., Fremont, CA, USA). IOP was measured using TONOVET (M.E. Technica, Tokyo, Japan) under topical anesthesia. Three measurements were taken, from which the mean IOP was recorded. Immediately after surgery and after examination on postoperative days 1, 2, and 5, 0.2 mL 10 µM PD0325901 or 0.1% DMSO was injected under the bleb.

### Histologic evaluation

The rabbits were euthanized by intravenous injection of secobarbital (150 mg/kg body weight) at 7 days after surgery. Frozen sections were made as described previously^43^. After enucleation, the eyes were immersed in 4% paraformaldehyde and fixed for 24 h. Then the eyes were dehydrated in 30% sucrose for 24 h. Subsequently, the eyes were embedded in optimal cutting temperature compound (Tissue-Tek; Sakura Finetek, Tokyo, Japan). The sections were stained with HE and EVG. The evaluation of fibrosis was conducted in a masked manner.

### Statistical analysis

The results are presented as means ± standard deviations. Statistical analyses were performed using R ver. 4.2.1 (The R Foundation for Statistical Computing, Vienna, Austria). The results of CCK-8 were analyzed using Dunnett's test, while the results of the other experiments were analyzed using t-tests. P < 0.05 was considered statistically significant.

### Supplementary Information

Supplementary Figures.

## Data Availability

All data generated or analyzed in this study are available from the corresponding author upon reasonable request.
