# Supplementary material for: A MEK inhibitor arrests the cell cycle of human conjunctival fibroblasts and improves the outcome of glaucoma filtration surgery
Source: Sci Rep. 2024 Jan 22;14:1871. doi: 10.1038/s41598-024-52359-y (PMC10803501; doi:10.1038/s41598-024-52359-y)
Supplement: Supplementary file 1 — Supplementary Figures. [file 41598_2024_52359_MOESM1_ESM.pdf]

a

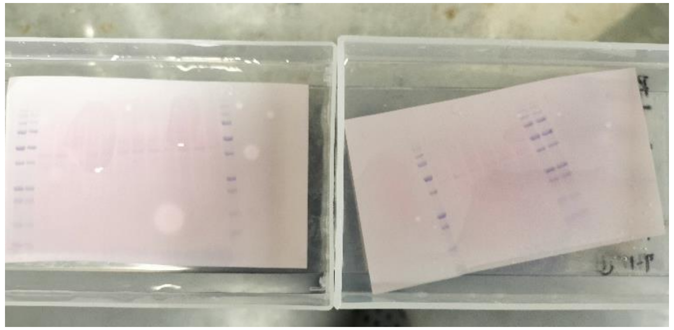

b

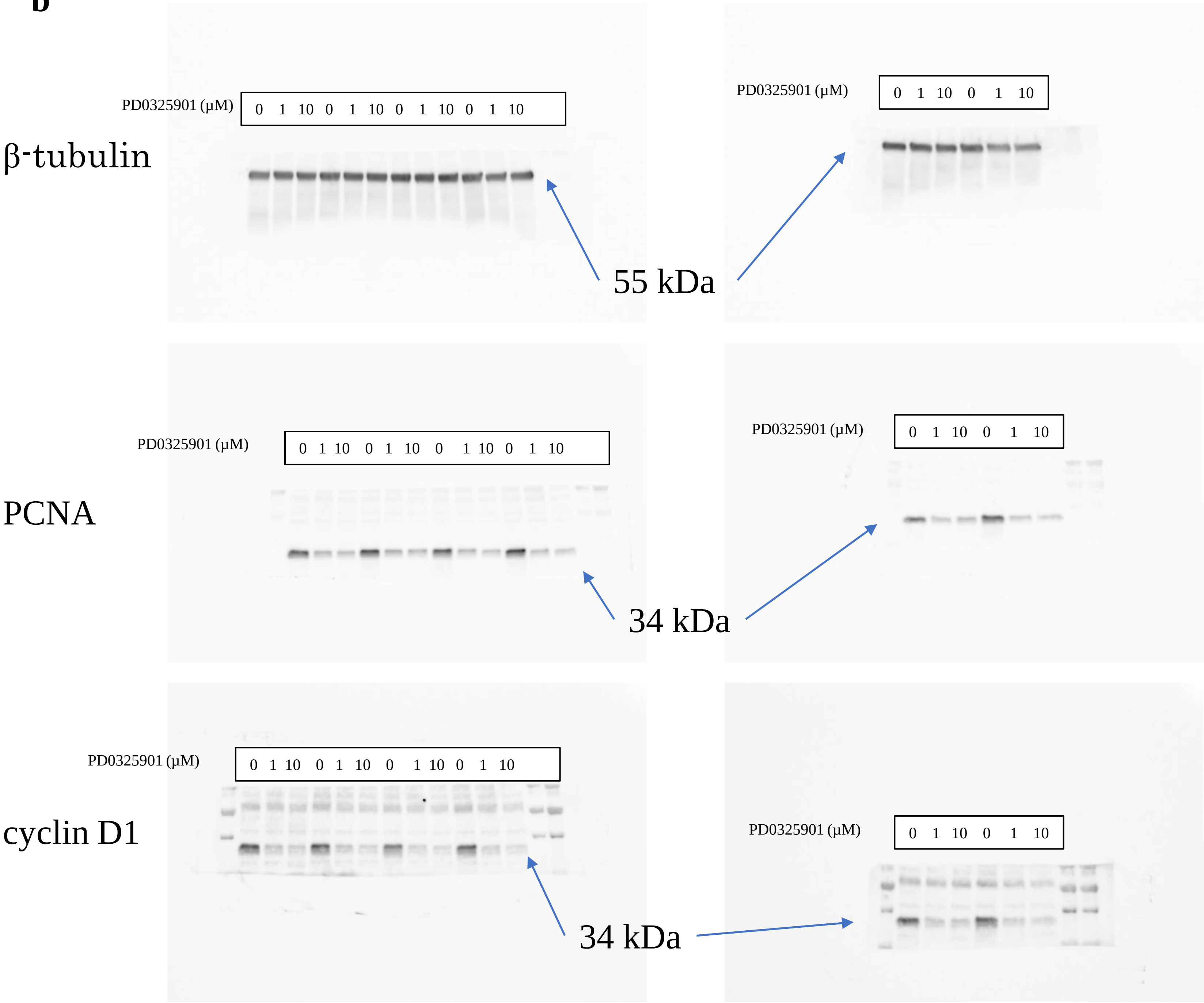

Supplemental Fig. 1

(a) Original membranes stained with Ponceau S. Molecular size markers are shown from top to bottom: 250, 150, 100, 75, 50, 37, 25, 20, 15, and 10 kDa.

(b) The original blots of β-tubulin, PCNA and cyclin D1.

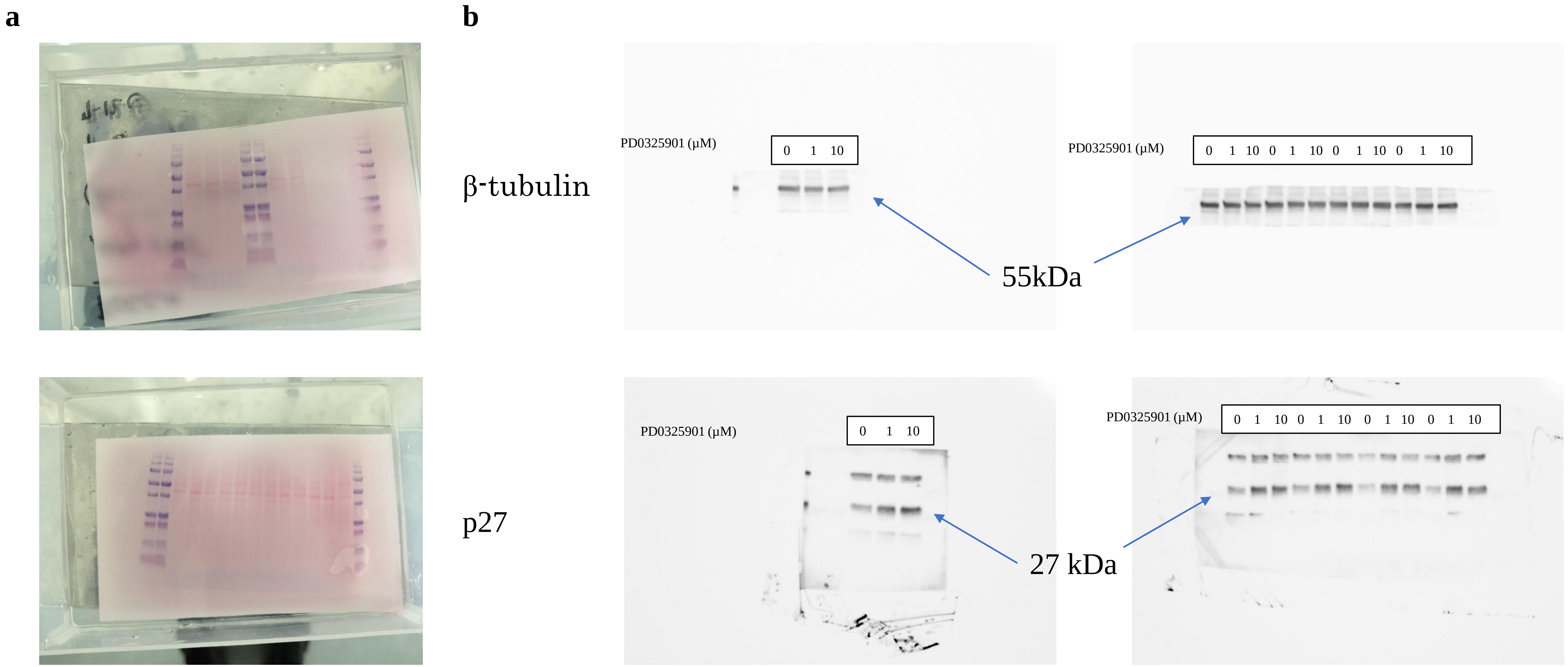

Supplementary Fig. 2

(a) Original membranes stained with Ponceau S. Molecular size markers are shown from top to bottom: 250, 150, 100, 75, 50, 37, 25, 20, 15, and 10 kDa. The lanes shown on the right side of the upper photo were cut before hybridization with antibodies because the sample was not correctly applied to the lanes.

(b) The original blots of β-tubulin and p27.
